# Implementing collaborative care to reduce depression for rural native American/Alaska native people

**DOI:** 10.1186/s12913-019-4875-6

**Published:** 2020-01-13

**Authors:** Deborah J. Bowen, Diane M. Powers, Joan Russo, Robert Arao, Erin LePoire, Earl Sutherland, Anna D. H. Ratzliff

**Affiliations:** 1 University of Washington, A204, 1959 NE Pacific Street, Seattle, WA 98195 USA; 2 Bighorn Valley Health Center, 10 4th Street W, Hardin, MT USA

**Keywords:** Native American/Alaska native, Depression, Collaborative care, Implementation process

## Abstract

**Background:**

The purpose of this study was to identify the effects of Collaborative Care on rural Native American and Alaska Native (AI/AN) patients.

**Methods:**

Collaborative Care was implemented in three AI/AN serving clinics. Clinic staff participated in training and coaching designed to facilitate practice change. We followed clinics for 2 years to observe improvements in depression treatment and to examine treatment outcomes for enrolled patients. Collaborative Care elements included universal screening for depression, evidence-based treatment to target, use of behavioral health care managers to deliver the intervention, use of psychiatric consultants to provide caseload consultation, and quality improvement tracking to improve and maintain outcomes. We used t-tests to evaluate the main effects of Collaborative Care and used multiple linear regression to better understand the predictors of success. We also collected qualitative data from members of the Collaborative Care clinical team about their experience.

**Results:**

The clinics participated in training and practice coaching to implement Collaborative Care for depressed patients. Depression response (50% or greater reduction in depression symptoms as measured by the PHQ-9) and remission (PHQ-9 score less than 5) rates were equivalent in AI/AN patients as compared with White patients in the same clinics. Significant predictors of positive treatment outcome include only one depression treatment episodes during the study and more follow-up visits per patient. Clinicians were overall positive about their experience and the effect on patient care in their clinic.

**Conclusions:**

This project showed that it is possible to deliver Collaborative Care to AI/AN patients via primary care settings in rural areas.

## Contributions to the literature

- Clinics serving primarily AI/AN patients are interested in improvements to treat depression by implementing Collaborative Care Management.
- Clinics serving AI/AN patients Native American can change their practices to deliver Collaborative Care Management to treat depression.
- AN/AI patients do as well as Caucasian patients when Collaborative Care management is delivered

## Background

Depression is a chronic and often debilitating disease and the leading cause of disability worldwide [1]. Depression often co-occurs with chronic medical conditions and substantially worsens associated health outcomes [2]. When depression is not effectively treated, it can impair self-care and participation in needed medical care, increase mortality, substantially increase overall health care costs, and decrease work productivity and economic well-being [3].

Many Native American/Alaska Native (AI/AN) communities face a high burden from the effects of depression. While rates of depression in AI/AN communities vary across communities and tribal settings [4, 5], substantial evidence with reasonable methodological rigor suggests AI/AN communities face a high burden of psychological morbidity [5]. AI/AN communities include over 560 federally recognized tribes with unique backgrounds and current experiences that may impact depression (e.g., cultural connections, experiences of racial discrimination, trauma, poverty). Historical and current losses experienced by AI/AN communities may affect both mental and physical health [6]. Combined with lack of access to mental health services, depression is a major health problem that needs increased intervention and treatment [7].

Primary care represents an important strategy for treating depression in populations facing health disparities with limited or no access to specialty mental health care. The prevalence of psychiatric disorders in Federally Qualified Health Centers (FQHCs) is substantially higher than in private primary care settings [8] because FQHCs, commonly referred to as the nation’s medical “safety net”, serve a disproportionate number of low-income patients [9]. However, the vast majority of FQHCs do not have psychiatrists or psychologists practicing on site [10]. In 2012, there was 1 psychiatrist per 49,764 primary care clinic patients and 1 psychologist per 43,505 patients [11]. Moreover, the National Academy of Medicine describes the linkages between primary care clinics and Community Mental Health Centers (the nation’s mental health setting safety net) as weak [12–14]. Innovative solutions are needed to address this problem.

Collaborative Care Management (CoCM) is a practice-based system of care designed to integrate treatment for common mental health disorders (e.g. depression, anxiety) into primary care settings using principles of chronic disease management [15]. This approach uses existing pharmacologic and psychotherapeutic treatments in a new way through a team-based approach [16–18]. Over the past 25 years, more than 80 randomized controlled research trials have established a robust evidence base for CoCM [2, 17, 18]. CoCM treatment is provided by a primary care-based team, including: 1) the primary care provider (PCP), who prescribes medications when they are part of the treatment plan and who coordinates medical and mental health treatment, 2) a psychiatric consultant, who provides indirect care through systematic case review rather than ad hoc consultation, and 3) a behavioral health care manager (CM) who supports measurement-based, treatment-to-target and provides evidence-based, brief, structured psychotherapy when that is part of the treatment plan. Behavioral health care managers are most commonly Master’s level licensed clinical social workers and licensed counselors (i.e., master’s level therapists). Some clinics share behavioral health care management responsibilities with medical assistants or community health workers under the supervision of the licensed provider. Behavioral health care manager responsibilities include: 1) screening and identification, 2) assisting with differential diagnosis and treatment planning, 3) patient engagement and education, 4) pro-active follow-up focusing on treatment adherence and effectiveness, 5) providing brief evidence-based psychotherapy such as, Behavioral Activation, and Problem-Solving Treatment adapted for primary care, 6) regular (usually weekly) review of all patients who are not improving as expected with a psychiatric consultant, and 7) facilitation of communication between the PCP and the psychiatric consultant. Rather than functioning as a separate, parallel provider co-located in the primary care setting, the behavioral health care manager and PCP use a shared care plan and collaborate on treatment planning and treatment changes. Psychiatric consultants (usually psychiatrists and sometimes psychiatric nurse practitioners) provide recommendations to the PCP and CM, focusing on diagnosis, treatment planning for new patients, and changes to treatment for patients who are not improving after 10–12 weeks with the current treatment plan. Primary care providers see patients and prescribe medications, monitoring potential side effects through ongoing visits with support from the CM.

Recent publications and reports have called for research into the implementation of better depression treatments for AI/AN populations by adapting and implementing existing programs used to treat depression in the general population [19–21]. For example, papers have documented successful use of cognitive-behavioral therapy for AI/AN people [21]. Yet, none of these studies have focused on clinic-level implementation of depression treatment, which holds promise for improving the health and well-being of communities served by these clinics as well as individual patients. A briefing book published by the Indian Health Service (IHS) recommends implementing adapted versions of evidence-based depression treatment focused on patients, but there is no discussion about clinic-level implementation and few implementations have engaged primary care [20]. Some smaller studies have used principles of CoCM to improve depression treatment, demonstrating that CoCM is feasible in AI/AN settings, although these have not been clinic or system-wide efforts to scale better care for AI/AN people [21]. These studies indicate the promise of CoCM to more effectively treat depression among AI/AN people.

The Social Innovation Fund, a public-private partnership between the federal government and philanthropy, supported an initiative (SIF-CoCM) to implement and evaluate outcomes of CoCM in 8 rural FQHCs in the Western US. A previous paper reports positive clinical outcomes: 47% of patients experienced depression response (50% reduction of symptoms from baseline to last measurement) and 24% experienced depression remission (near absence of depression symptoms from baseline to last measurement) during this period [22]. Three of these FQHCs treat a large number of AI/AN patients, providing an opportunity to compare the effects of CoCM on depression outcomes between AI/AN, White patients and patients of other ethnic backgrounds. Identification of the potential usefulness of CoCM to treat AI/AN patients with depression holds promise for population-based, larger scale implementation in a range of primary care settings that treat AI/AN people.

The purpose of this study was to compare the effects of implementing CoCM on depression outcomes in AI/AN patients as compared with White patients and patients of other ethnic backgrounds at these three clinics. Here we describe the sample, the overall effects of implementation on depression response and remission among AI/AN versus White and other patients, the variation in response and remission associated with demographic variables and processes of care, and the reactions of providers to the implementation process.

## Methods

### Design of this project

The design of this implementation initiative details how participating clinics were selected, practice changes necessary for effective CoCM implementation in limited-resource settings, and characteristics of participating primary care clinics are described elsewhere [23]. Briefly, evaluation of the SIF-CoCM initiative used an observational study design to measure primary outcomes: depression response and depression remission. Response is defined as a 50% or greater reduction in symptom severity, as measured by the PHQ-9, from baseline to last measurement. Remission is defined as a score of five or less on the PHQ-9 at last measurement. These definitions, based on the psychometric properties of the PHQ-9 [24], are HEDIS measures commonly used in primary care practice and evaluation [25]. Process of care metrics shown in prior analyses to predict better clinical outcomes, including the number of patient contacts within the first 4 weeks of treatment and the proportion of patients whose care is informed by consultation with the psychiatric expert, were also calculated [17, 26]. Eight FQHCs were originally recruited for the SIF project; three of these clinics had sizable AI/AN samples and are the focus of this paper. The average number of active patients across all three clinics in the year before SIF-CoCM started was 4083. Among the three clinics, the proportion of AI/AN patients as % of patients enrolled in CoCM, was 3.3% at Clinic C, 51.2% at Clinic F, and 71.8% at Clinic G. The study was reviewed by the University of Washington Institutional Review Board. This Board determined that this was not human subjects research and all consent was therefore waived.

### Clinic descriptions

#### Clinic C

Clinic C has been an FQHC for nearly 30 years, serving a five county region covering more than 10,000 mile^2^ in the Rocky Mountain West. They operate six clinical sites offering medical, dental, behavioral health, and pharmacy services serving about 15,000 patients annually. Forty-three percent of their patients live at or below 100% of the federal poverty level and nearly one quarter are uninsured. This clinic came to the SIF-CoCM implementation initiative with a substantial history of quality improvement experience, including population-based care management for chronic medical illnesses, and stable leadership who had been with the organization for many years. Contemporaneous with implementing SIF-CoCM the clinic launched a family medicine residency program which significantly impacted primary care workflows and the number of providers involved in primary care delivery.

#### Clinic F

Clinic F is located in the upper Great Plains, and came into existence about 18 months before they applied to participate in SIF-CoCM. At the time of SIF-CoCM launch, the clinic offered primary care services at one clinic location. By the end of SIF-CoCM, they had expanded to two additional physical locations in nearby communities and expanded their services to include pharmacy services and medication assisted addiction treatment.

#### Clinic G

Alaska has numerous tribal health organizations that operate healthcare facilities, funded all or in part by the Indian Health Service but controlled by the local Alaska Native people. Clinic G is one of these clinics operating in a geographically remote area serving the AI/AN community and other residents of a small town as well as several isolated Alaska Native villages in their geographic catchment area. Many organizations in rural Alaska, including this clinic, struggle to recruit healthcare providers. Providers are typically offered a relocation incentive if they stay 2 years and may qualify for various types of student debt relief. However, providers rarely stay more than 2 years. During the course of SIF-CoCM, there was significant turnover among clinic providers and leadership.

##### Data sources

The data source for primary and secondary patient-level outcomes was the Care Management Tracking System (CMTS) [26], a HIPPA-compliant, web-based disease management registry designed to facilitate CoCM delivery. The registry tracks patient-level clinical outcomes for depression and comorbid conditions (e.g., anxiety) and clinic-level processes of care. Depression symptoms were measured with the PHQ-9 depression symptom rating scale [24] at treatment initiation (baseline) and subsequent contacts.

To better understand the implementation process, providers at participating clinics completed a survey of their experiences with SIF-CoCM 18 months following the program launch. The purpose was to assess the benefits and burdens of implementing CoCM in these rural clinics.

##### Evaluation measures

Depression symptoms and severity were assessed with the PHQ-9, a well-validated self-report measure commonly used in primary care and other settings for screening and tracking improvement over time [24, 27]. A single item from the PHQ-9 was used to assess suicidal ideation, defined as any value larger than “0”. This item has been shown to predict suicide attempts and is a moderate predictor of subsequent suicide death [28]. Processes of depression care relevant to the full implementation of CoCM were assessed as part of the CMTS and were calculated as described here: average number of follow-up visits in a single depression episode, type of visit (in-clinic versus telephone), proportion of patients with a follow-up contact within 4 weeks of intake/baseline, percent of patients completing follow-up (2+ contacts), percent of patients engaged in treatment ≥12 and > 40 weeks, and percent of patients discussed with the psychiatric consultant at least once.

We conducted a survey of CoCM provider roles (PCP, CM, psychiatric consultant) via an online web system called REDCap, which sends an email to potential participants with a link to a survey and asks them to participate. The survey contained closed-ended questions on provider satisfaction, quality of care, and open-ended questions for providers to comment on the most positive and negative aspects of the implementation process. Questions included appraisal of the effectiveness of CoCM in improving access to care and quality of care for their patients, whether or not they received the support they needed from the clinic to be successful, level of burnout, and demographic information such as professional training, years of clinical experience, and length of employment at the participating clinic.

##### Analytic plan

Patients were stratified into three self-reported racial/ethnic groups for analyses: AI/AN, White, and Other (e.g. Asian, African American). Demographic, clinical, and process of care variables were examined for the racial/ethnic groups using chi-square analyses for categorical variables and Analyses of Variance for continuous variables. Significance levels were set at 0.05 and data were reported with 95% confidence levels. Missing data and unknown data were excluded from analyses. In the event of a significant 3-group test, three planned comparisons were used to determine which pairs of groups contributed to the significance. The goal of our analyses was to determine if race/ethnicity was significantly related to our two outcomes, depression response and remission. We tested the significance of odds ratios for AI/AN patients, in 3 hierarchical logistic regression models. The first model included only demographic variables; the second model added clinic/site to the model. The third model included demographic variables, clinic, and the statistically significant clinical (maximum PHQ-9 score during the treatment episode, baseline PHQ-9) and process variables (treatment duration in months, prior episodes of depression treatment in the clinic, number of follow-up visits, psychiatrist notes, and first follow up within a month of intake). Process variables not significantly related to the outcomes were removed from the model and the model was refit. We selected the reference group based in general on the largest comparator group by sample size. For the open-ended analyses we simply reviewed all answers to questions asked on the survey and recorded the answers.

The open-ended data and responses were very simple and short, due to the method of collection using fill-in blanks. We used very simple thematic coding to better see patterns in the open-ended data from the surveys. We identify quotations to support our choice of themes using a label about which staff person provided the quote (CM for Care manager and PCP for primary care provider). Quotes are presented from all three AI/AN serving clinic staff groups, to illustrate the findings.

We used the TIDieR checklist to improve the reporting of this intervention [29]. We used guidelines specifically for indigenous people to guide the reporting of these findings [30].

## Results

Our 3-clinic sample of 1993 patients included 345 AI/AN patients (17%), 74% White patients (*n* = 1473), and 175 patients designated as “Other” (9%). Table 1 presents the demographic variables utilized in the regression models stratified by the ethnic-racial groups from the three clinics. AI/AN patients were significantly more likely to be women than either Whites or patients with Other ethnicities. Age differences were due to the “Other” group being more likely to be younger than the White group, which did not differ from the AI/AN group. Categorized depression severity measures (Baseline PHQ-9, Last PHQ-9, and Maximum PHQ-9) were all significantly different between the ethnic groups. Planned comparisons showed the same pattern for all measures, with the AI/AN group reporting significantly lower depression severity than the White and Other groups on all these measures. The race/ethnicity groups had slightly different total numbers of prior episodes of depression treatment.

**Table 1:** Background variables of SIF-CoCM AI/AN sample

category (*N* = 1993)	AI/AN (*N* = 345)	White (*N* = 1473)	Other (*N* = 175)	Total (*N* = 1993)	*p*-value^ǂ^	1 vs 2	2 vs 3	1 vs 3
Gender (*n* = 1979)								
Women	248 (71.9)	944 (64.2)	94 (57.7)	1286 (65.0)	0.003	^***^		^***^
Men	97 (28.1)	527 (35.8)	69 (42.3)	693 (35.0)				
Age (*n* = 1976)								
18–34	157 (45.5)	603 (41.2)	88 (52.4)	848 (42.9)	< 0.001		^***^	
35–54	130 (37.7)	536 (36.6)	57 (33.9)	723 (36.6)				
55–74	50 (14.5)	316 (21.6)	23 (13.7)	389 (19.7)				
75+	8 (2.3)	8 (0.5)	0 (0.0)	16 (0.8)				
Initial patient PHQ (*n* = 1993)								
< 10	73 (21.2)	212 (14.4)	24 (13.7)	309 (15.5)	< 0.001	^***^		^***^
10–14	109 (31.6)	398 (27.0)	49 (28.0)	556 (27.9)				
15–19	100 (29.0)	415 (28.2)	55 (31.4)	570 (28.6)				
20–27	63 (18.3)	448 (30.4)	47 (26.9)	558 (28.0)				
Last PHQ (*n* = 1993)								
< 10	178 (51.6)	638 (43.3)	69 (39.4)	885 (44.4)	0.003	^***^		^***^
10–14	77 (22.3)	344 (23.4)	36 (20.6)	457 (22.9)				
15–19	60 (17.4)	255 (17.3)	41 (23.4)	356 (17.9)				
20–27	30 (8.7)	236 (16.0)	29 (16.6)	295 (14.8)				
One	282 (81.7)	1232 (83.6)	160 (91.4)	1674 (84.0)	0.005		^***^	
Two	57 (16.5)	186 (12.6)	12 (6.9)	255 (12.8)				
Three or more	6 (1.7)	55 (3.7)	3 (1.7)	64 (3.2)				

^***^ Significant at 0.05 level using Tukey method

^ǂ^ *p*-values excludes unknowns/missing in calculation

Table 2 shows the depression outcomes and depression severity measures after treatment stratified by the three groups. AI/AN patients reported the lowest level of depression severity at both baseline and the last PHQ-9 measurement. As a group, they also had significantly less suicidal ideation at both baseline and last measurement. Although depression remission rates did not vary significantly between the groups (21.9% overall), depression response was greatest in the AI/AN patients (52.8%) in comparison to the White (44.8%) and the “Other” (40.6%) groups.

**Table 2:** Depression Outcomes in SIF-CoCM AI/AN samples

	AI/AN (*N* = 345)	White (*N* = 1473)	Other (*N* = 175)	Total (*N* = 1993)	*p*-value^ǂ^	1vs2	2vs3	1vs3
Baseline PHQ-9 (mean, std., *n* = 1993)	13.9 (6.2)	15.8 (6.1)	16.0 (5.6)	15.5 (6.1)	< 0.001	^***^		^***^
Last PHQ-9 (mean, std., *n* = 1993)	10.0 (6.6)	11.4 (7.1)	12.0 (7.3)	11.2 (7.1)	< 0.001	^***^		^***^
^a^PHQ-Remission (*n* = 1993)	87 (25.2%)	311 (21.1%)	38 (21.7%)	436 (21.9%)	0.252			
^b^PHQ-Response (*n* = 1993)	182 (52.8%)	660 (44.8%)	71 (40.6%)	913 (45.8%)	0.010	^***^		^***^
Suicidal Ideation at Baseline (*n* = 1501)	106 (31.5%)	434 (41.7%)	56 (45.5%)	596 (39.7%)	0.002	^***^		^***^
Suicidal Ideation at Last PHQ-9 (*n* = 1509)	70 (22.7%)	288 (26.3%)	31 (29.8%)	389 (25.8%)	0.272			

^***^ Significant at 0.05 level using Tukey method

^ǂ^ *p*-values excludes unknowns/missing in calculation

^a^PHQ-Remission: Last PHQ-9 < 5

^b^PHQ-Response: (50% decrease or PHQ-9 < 10 at last follow-up)

Table 3 presents the processes of care stratified by the three racial/ethnic groups. A few CoCM evidence-based processes of care showed significant but slight differences among the three groups of patients. For example, patients in the Other category had higher numbers of in-clinic follow-up visits compared to the other two groups, as well as more follow-up during the first 4 weeks of treatment compared to the other groups. AI/AN patients had lower follow-up rates compared to White and Other patients. No other differences were significant.

**Table 3:** Processes of Care for AI/AN, White, and Other patients

	AI/AN	White	Other	Total	*p*-value
Total number of contacts	1669	19,547	2617	23,833	
Mean Number Contacts (95% CI)	4.7 (4.2,5.2)	5.2 (5.0,5.4)	5.4 (4.9,5.9)	5.2 (5.0,5.3)	-ns
In-Clinic Sessions (%)	82.7	82.4	87.3	83.0	< 0.0001
Phone Sessions (%)	16.2	16.3	11.7	15.8	< 0.0001
% first follow-up w/in < 4 weeks	54.4	59.1	63.7	59.2	0.0177
% completing follow-up (2+ contacts)	59.7	67.6	66.5	66.9	0.0066
% engaged in treatment ≥12 weeks	5.8	3.9	3.3	4.0	0.1178
% engaged in treatment > 40 weeks	0.0%	0.4%	0.0%	0.3%	0.1984
% at least 1 psych consult	85.1	88.8	73.0	86.9	< 0.0001

Tables 4, 5, and 6 show the logistic regression models for the depression remission and response outcomes, respectively. In comparison to the reference group of White patients, AI/AN patients were more likely to have a depression response to treatment (OR = 1.4, 95% CI = 1.1–1.7) in the demographic model (Table 4). Adding clinic to the model (Table 5) resulted in race/ethnicity no longer being significantly related to depression response (*p* = .14). Adding process of care variables to the model further reduced the significance of AI/AN in the model (*p* = .52). In the full model, clinic, severity of the maximum PHQ-9 (OR = 0.8, 95% CI = 0.8–0.8), treatment duration (OR = 1.1, 95% CI = 1.1–1.2), treatment episodes (OR = 0.5, 95% CI = 0.4–0.8), and total number of follow-ups contacts (OR = 2.4, 95% CI = 1.7–3.5 for 2–3 visits; OR = 4.9, 95% CI = 3.3–7.3 for 4–7 visits) were all statistically significant. Patients who experienced depression response were more likely to have a lower maximum PHQ-9, longer treatment duration, fewer episodes of treatment, and more follow-up visits.

**Table 4:** Demographics Only as predictors of Depression outcomes in SIF-CoCM AI/AN clinics

Category	Response					Remission				
	N (%)	Odds Ratio	LowerCL	UpperCL	*p*-value	N (%)	Odds Ratio	LowerCL	UpperCL	*p*-value
Age										
age 18–34 (referent)	357 (42.1)	–	–	–	–	166 (19.6)	–	–	–	–
age 35–54	328 (45.4)	1.1	0.9	1.4	0.192	153 (21.2)	1.1	0.9	1.4	0.350
age 55–74	210 (54.0)	1.6	1.3	2.1	< 0.001	108 (27.8)	1.6	1.2	2.1	0.001
age > =75	13 (81.3)	5.4	1.5	19.3	0.009	8 (50.0)	4.0	1.5	11.0	0.006
Race										
White (referent)	660 (44.8)	–	–	–	–	311 (21.1)	–	–	–	–
AI/AN	182 (52.8)	1.4	1.1	1.7	0.009	87 (25.2)	1.2	0.9	1.6	0.124
Other	71 (40.6)	0.9	0.6	1.2	0.462	38 (21.7)	1.2	0.8	1.7	0.424
Gender										
Male (referent)	307 (44.3)	–	–	–	–	135 (19.5)	–	–	–	–
Female	600 (46.7)	1.3	1.0	1.6	0.042	299 (23.3)	1.3	1.0	1.6	0.042

Initial predictors entered prior to variable selection: age, race, gender, treatment duration (months), number of episodes, number of follow-ups, first follow-up within 31 days, at least one psychiatric consultation note

**Table 5:** Demographics and clinic as predictors of Depression outcomes in SIF-CoCM AI/AN clinics

Category	Response					Remission				
	N (%)	Odds Ratio	LowerCL	UpperCL	*p*-value	N (%)	Odds Ratio	LowerCL	UpperCL	*p*-value
Age										
age 18–34 (referent)	357 (42.1)	–	–	–	–	166 (19.6)	–	–	–	–
age 35–54	328 (45.4)	1.1	0.9	1.4	0.269	153 (21.2)	1.1	0.9	1.4	0.435
age 55–74	210 (54.0)	1.6	1.3	2.0	< 0.001	108 (27.8)	1.6	1.2	2.1	0.001
age > =75	13 (81.3)	4.4	1.2	15.8	0.024	8 (50.0)	3.3	1.2	9.0	0.023
Race										
White (referent)	660 (44.8)	–	–	–	–	311 (21.1)	–	–	–	–
AI/AN	182 (52.8)	0.8	0.6	1.1	0.138	87 (25.2)	0.7	0.5	1.1	0.113
Other	71 (40.6)	0.8	0.6	1.1	0.203	38 (21.7)	1.1	0.7	1.6	0.721
Gender										
Male (referent)	307 (44.3)	–	–	–	–	135 (19.5)	–	–	–	–
Female	600 (46.7)	1.1	0.9	1.3	0.279	299 (23.3)	1.3	1.0	1.6	0.032
Site										
Clinic C (referent)	294 (19.3)					638 (41.9)				
Clinic F	72 (34.8)	3.0	2.1	4.2	< 0.001	137 (66.2)	2.4	1.7	3.5	< 0.001
Clinic G	70 (26.7)	1.8	1.3	2.6	0.001	138 (52.7)	1.8	1.2	2.7	0.004

Initial predictors entered prior to variable selection: age, race, gender, treatment duration (months), number of episodes, number of follow-ups, first follow-up within 31 days, at least one psychiatric consultation note

**Table 6:** Demographics, clinic, and process variables as predictors of Depression outcomes in SIF-CoCM AI/AN sites

Level	Response					Remission				
	N (%)	Odds Ratio	LowerCL	UpperCL	*p*-value	N (%)	Odds Ratio	LowerCL	UpperCL	*p*-value
Age										
age 18–34 (referent)	357 (42.1)	–	–	–	–	166 (19.6)	–	–	–	–
age 35–54	328 (45.4)	0.9	0.7	1.2	0.600	153 (21.2)	0.9	0.7	1.2	0.560
age 55–74	210 (54.0)	1.3	0.9	1.7	0.176	108 (27.8)	1.3	0.9	1.8	0.182
age > =75	13 (81.3)	1.8	0.4	7.8	0.453	8 (50.0)	1.7	0.5	5.4	0.363
Race										
White (referent)	660 (44.8)	–	–	–	–	311 (21.1)	–	–	–	–
AI/AN	182 (52.8)	1.2	0.7	1.8	0.523	87 (25.2)	0.9	0.6	1.4	0.654
Other	71 (40.6)	0.9	0.6	1.3	0.485	38 (21.7)	1.4	0.9	2.2	0.177
Gender										
Male (referent)	307 (44.3)	–	–	–	–	135 (19.5)	–	–	–	–
Female	600 (46.7)	1.1	0.8	1.4	0.605	299 (23.3)	1.3	1.0	1.8	0.036
Site										
Clinic C (referent)	294 (19.3)	–	–	–	–	638 (41.9)	–	–	–	–
Clinic F	72 (34.8)	3.4	2.1	5.5	< 0.001	137 (66.2)	2.2	1.5	3.4	< 0.001
Clinic G	70 (26.7)	0.9	0.6	1.5	0.779	138 (52.7)	1.0	0.6	1.7	0.922
Severity of Max PHQ										
continuous	–	0.8	0.8	0.8	< 0.001	–	0.8	0.8	0.9	< 0.001
Treatment Duration (months)										
continuous	–	1.1	1.1	1.2	< 0.001	–	1.1	1.0	1.1	0.009
Total Treatment Episodes										
1 episode (referent)	795 (47.5)	–	–	–	–	382 (22.8)	–	–	–	–
two or more episodes	118 (37.0)	0.5	0.4	0.8	< 0.001	54 (16.9)	0.7	0.5	1.0	0.035
Total FU										
< =1 FU (referent)	95 (32.4)	–	–	–	–	40 (13.7)	–	–	–	–
2–3 FU	208 (45.5)	2.4	1.7	3.5	< 0.001	87 (19.0)	1.8	1.1	2.8	0.011
4–7 FU	268 (62.0)	4.9	3.3	7.3	< 0.001	149 (34.5)	4.4	2.8	7.0	< 0.001

Initial predictors entered prior to variable selection: age, race, gender, treatment duration (months), number of episodes, number of follow-ups, first follow-up within 31 days, at least one psychiatric consultation note

Race/ethnicity was not related to remission in any of the three models. In the full model, depression remission was related to clinic, female gender (OR = 1.3, 95% CI = 1.0–1.8), lower maximum PHQ-9 (OR = 0.8, 95% CI = 0.8–0.9), longer duration of treatment (OR = 1.1, 95% CI = 1.0–1.1), having fewer episodes of treatment (OR = 0.7, 95% CI = 0.5–1.0), and having more follow up contacts (OR = 1.8, 95% CI = 1.1–2.8 for 2–3 visits; OR = 4.4, 95% CI = 2.8–7.0 for 4–7 visits).

The provider survey further elucidated the implementation process in these three clinics. We surveyed the three types of providers who comprise the CoCM team at each clinic: primary care providers (PCPs *n* = 25), psychiatric consultants (n = 2), and care managers (*n* = 13).

Generally, primary care providers reported they found key aspects of CoCM to be very helpful for their clinical practice. In these three clinics, survey data indicated that 96% of primary care providers and both psychiatric consultants found primary care and behavioral specialists working together “very helpful” to manage patients with depression. When asked to elaborate on what principles of CoCM best fit into their organizational culture, many chose patient-centered care team.

“The patient-centered approach allows for a closer degree of collaboration between the patient’s medical and behavioral health treatments”- PCP Clinic C

“[CoCM] provides collaborative effort between counselors, psychiatry and primary care physicians” –PCP Clinic G

“We strive to maintain effective teamwork across the disciplines” PCP- Clinic F

“At our clinic, the medical providers, behavioral health providers and patients work collaboratively to identify, address, and treat depression” –PCP Clinic F

Fewer care managers (62%) across the three clinics reported that having primary providers and behavioral health specialists work together to manage patients with depression was very helpful. When asked about which CoCM principle fit *least* within their organization many care managers chose accountable care and patient centered team care.

– “Accountable Care fits least with the organizational culture of our clinic because there is not a shared accountability for the health care needs and outcomes of patients. The care managers are almost always the ones held accountable for the outcomes of patients. When a patient is not getting better, or their PHQ-9 is not improving it always comes down to what we are doing wrong as care managers. It's incredibly discouraging because we are the member of the team with the least amount of education and ability to make changes.” – CM Clinic C

– “We still struggle with defining which team member does what, how to best communicate [CoCM] model to the PCP’s, and their role in the patient’s treatment” -CM Clinic C

– “The team atmosphere is good but could always be improved. The clinic sometimes operates in separate entities due to the clinic layout.” –CM Clinic C

– “Measurement based treatment to target is a weaker area, particularly in terms of routine team coordination” –CM Clinic G

The majority of clinicians of all types (62%) indicated CoCM made definite improvements in patient care in their clinic. When asked to comment on what they liked most about CoCM, many participants responded with how CoCM benefitted their clinic

“It is great to have [CoCM] available in the clinic. Most of our patients do not have insurance or resources to afford private therapists. It is affordable, easy to access and great care for our patients” – PCP Clinic C

– “I think [CoCM] is a great program when used with the right patient. I found that when the appropriate patient was put into the program, they really thrived and improved and I got a lot of good feedback from the patient about how much they liked the program and how quickly some of them improved and were able to get on with their lives . . . overall I think it is a great program/model with a lot of promise when used in the right setting. thanks for letting us be a part of it.” – PCP Clinic C

– “I like [CoCM’s] collaborative care approach and treating the person as a whole instead of bits and pieces – CM Clinic G (* identifies as AI/NA)

– “ I enjoy the integrated care system of [CoCM] and the holistic team approach. It creates the best care and treatment for the patients … and creates and environment where all providers from different perspectives work as a team to create accountability and holistic care.” –CM Clinic F (* identifies as AI/NA)–

– “[there is a] high level of competency, dedication to patients and efficiency of the [CoCM] team members”- PC Clinic C

However, care managers in these clinics expressed a lack of trust in using the PHQ-9 as the measurement of clinical success. In response to questions on principles of CoCM that are not working, many responded with measurement based treatment to target.

– “Our patient population and lack of local and state mental health resources make it difficult to accurately gauge our patients’ mental health using quantifiable measures. The PHQ-9 does not always do an adequate job of showing improvement in a patients’ behavioral health” – CM Clinic C

– “”such a focus on patient improvement, when patients do NOT improve, the program is targeted as not working, rather than expanding or adjusting patient care”- CM Clinic G

– “There are certain things that just can't be measured. I don't think the PHQ-9 accurately portrays how all people are feeling. I don't think one measurement should dictate how quickly someone moves through treatment.” – CM Clinic C

Finally, 90% of clinicians of all types reported improved work life quality following SIF-CoCM implementation.

## Discussion

Successful CoCM implementation at the clinic level resulted in positive effects for people of all ethnic groups. This is the largest study of CoCM implementation in clinics with large AI/AN populations and these findings demonstrate that CoCM improves depression outcomes for AI/AN people at equal levels when compared with White or other patients. In fact, the order of magnitude of the depression response was essentially similar for AI/AN patients compared to White patients within each clinic, and this effect held true, though variable, for all three clinics studied. This is an initial step toward using CoCM to improve outcomes among AI/AN patients on a larger scale.

These equivalent depression treatment outcomes were achieved by implementing the same CoCM protocol and using the same CoCM tools across all race/ethnic groups. These clinics compared favorably with clinics in the same implementation study that did not contain sizable numbers of AI/AN patients. In AI/AN serving clinics, 48.1% of patients experienced depression response and 25.1% experienced depression remission – outcomes very similar to those for AI/AN patients reported here. This indicates CoCM can be successfully implemented in a range of primary care clinics serving diverse patients and achieve comparable clinical outcomes.

All participating clinics were encouraged to adapt CoCM to meet the needs of their clinic settings and patients, while retaining key CoCM elements known to drive improved patient outcomes. We do not have information about adaptations the three clinics serving a large proportion of AI/AN patients made for the settings and populations, and this should be the focus of future research.

The depression response and remission rates reported here indicate AI/AN patients responded better to treatment than White patients at these clinics. Controlling for variation in clinic, analyses indicated this finding was not consistent across the three clinics. One clinic reported better depression response than the other two. The reason that occurred is not known. Large variation among clinics implementing CoCM is common but we know very little about why such variation occurs when clinics receive equivalent training and implementation support. Previous CoCM implementation projects suggest some variation might be due to the presence of a strong clinic champion and to other provider-driven variables [25]. The literature on implementation of innovation in AI/AN-serving clinics is sparse and to date has not contributed findings about why some clinics implement quality improvement practice change more successfully than others. Future efforts need to include more clinics serving AI/AN people and carefully measure variables at baseline and throughout implementation that could identify clinic-level predictors and correlates of implementation, thereby providing a better understanding of the underlying causes of clinic variation.

After controlling for overall clinic effect, processes of care remained related to both depression response and depression remission, suggesting that level of engagement in treatment still matters after controlling for clinic-level variation. This finding supports the impression of the implementation staff who coached clinics through practice change. These implementation coaches focused on evidence-based CoCM processes of care and patient clinical outcomes during monthly calls with clinics during the implementation support phase. Clinic staff and leaders were also taught to monitor these same processes of care to gauge implementation success.

The provider responses give depth to the patient data and provide insight into the process of implementation. Primary care providers were generally satisfied and supportive of the implementation process. This is important because PCPs are difficult to recruit to rural areas and clinics are eager to retain their workforce by providing a satisfying work environment. Behavioral health care managers reported mixed impressions regarding specific elements of CoCM, including the value of measurement based treat-to-target using the PHQ-9 and coordination of the CoCM treatment team. Using a measurement-based treatment-to-target approach is quite different from the training most licensed behavioral health care managers receive and traditional delivery of behavioral health services. Some CMs have difficulty with these differences. Also, behavioral health providers embedded in primary care often function in parallel to the PCP and without a significant amount of coordination. One of the key functions of the CM role is to serve as a facilitator for the entire treatment team (patient, PCP, and psychiatric consultant). This facilitation role may produce communication challenges for the CMs and some CMs may prefer to practice in a solo fashion.

As an evaluation of a real-world implementation, there are limitations to the generalizability of these results. These include the design of the project, with no control group, the low number of clinics involved, and the lack of explanatory variables to interpret clinic variability. Also, a larger number of clinics might have provided the sample size necessary to consider race/ethnicity of the provider as a predictive variable. In the current study there were not enough AI/AN providers to compare their outcomes to those of their colleagues in the three clinics studied.

The strengths of this study were the direct opportunity to compare AI/AN patients to White and Other patients from the same clinic, the relatively large sample size of AI/AN patients, and the real-life implementation of a complex quality improvement innovation in settings that serve AI/AN people. These strengths make this study’s findings important in understanding and informing future research and clinical care for depressed AI/AN primary care patients. They also provide hope for reducing depression among AI/AN patients in primary care and other settings where they receive general health care.

## Conclusions

In summary, we found that AI/AN-serving clinics were able to implement CoCM for depression, and were able to produce relatively high levels of depression response and remission in the patients they serve. Reactions of PCPs were generally positive, but reactions of behavioral health care managers were more mixed, indicating the complexity of this position in CoCM. Future research should focus on explaining and reducing the variation among clinics and supporting clinics in developing and implementing long-term financing models to promote sustainability. Additionally, implementing CoCM in more clinics with diverse resource availability, staffing patterns, and initial perspectives on CoCM may require different tools and supports to enable clinics to achieve their desired results.

## Data Availability

The datasets used and/or analyzed during the current study are available from the corresponding author on reasonable request.

## Abbreviations

CM: Behavioral health care manager
CMTS: Care Management Tracking System
CoCM: Collaborative Care Management
FQHC: Federally Qualified Health Center
NA/AN: Native American/Alaska Native
PC: Psychiatric Consultant
PCP: Primary Care Provider
SIF: Social Innovation fund
